# Case report: Different mechanisms of drug resistance in a synchronous multiple primary lung cancer patient after EGFR-TKI treatment

**DOI:** 10.3389/fonc.2022.977065

**Published:** 2022-09-29

**Authors:** Shaonan Xie, Qingyi Liu

**Affiliations:** Hebei Cancer Hospital, Shijiazhuang, China

**Keywords:** synchronous multiple primary lung cancer (SMPLC), epidermal growth factor receptor-tyrosine kinase inhibitors (EGFR-TKIs), resistance mechanism, T790 M mutation, MET amplification

## Abstract

Non-small-cell lung cancer (NSCLC) is the most common cancer in the world. In recent years, the incidence of synchronous multiple primary lung cancer (SMPLC) has gradually increased. Surgery is the preferred method to treat these patients. The management of SMPLC patients who cannot tolerate surgical treatment is controversial. We report a rare case in which a 70-year-old Chinese woman with no history of smoking had three primary lung adenocarcinoma lesions. Two lesions had epidermal growth factor receptor (*EGFR*) exon 19 deletion mutations, and one lesion had the L858R mutation. After first-generation EGFR–tyrosine kinase inhibitor (TKI) treatment, the three lesions all showed a good response until disease progression. After the corresponding drug treatments were given based on the different drug resistance mechanisms, good responsiveness was shown in each lessions. This case suggests that in the treatment of SMPLC, it is necessary to learn the molecular-biological information of each lesion due to the differences thereof, and a targeted treatment regimen should be developed on this basis.

## Introduction

Lung cancer is the cancer with the highest mortality rate in the world. With the advancement of modern medical technology, the 2-year survival rate of patients with non-small-cell lung cancer (NSCLC) has increased from 34% to 42% (1). With the growing application of computed tomography (CT), the incidence of synchronous multiple primary lung cancer (SMPLC) is increasing (2). Surgery is the main treatment for early lesions (3), but the diagnosis and treatment strategy of SMPLC is controversial for some patients who cannot be operated due to old age and underlying diseases.

Targeted therapy has been successfully and widely used in the treatment of lung cancer with driver gene mutations (4, 5). The incidence of epidermal growth factor receptor (*EGFR*) mutations in SMPLC patients is high (6–8). Approximately 45.8-76.0% of SMPLC patients in Asia have *EGFR* mutations, which means that EGFR–tyrosine kinase inhibitors (TKIs) can play a role in the diagnosis and treatment of these patients. However, the identities of the driver genes between individual tumors in SMPLC patients can differ by 72.0-92.1% (9), and tumors with targetable mutations are not representative of other tumors, which poses a challenge for the targeted therapy of SMPLC patients.

## Case presentation

**Figure 1:**
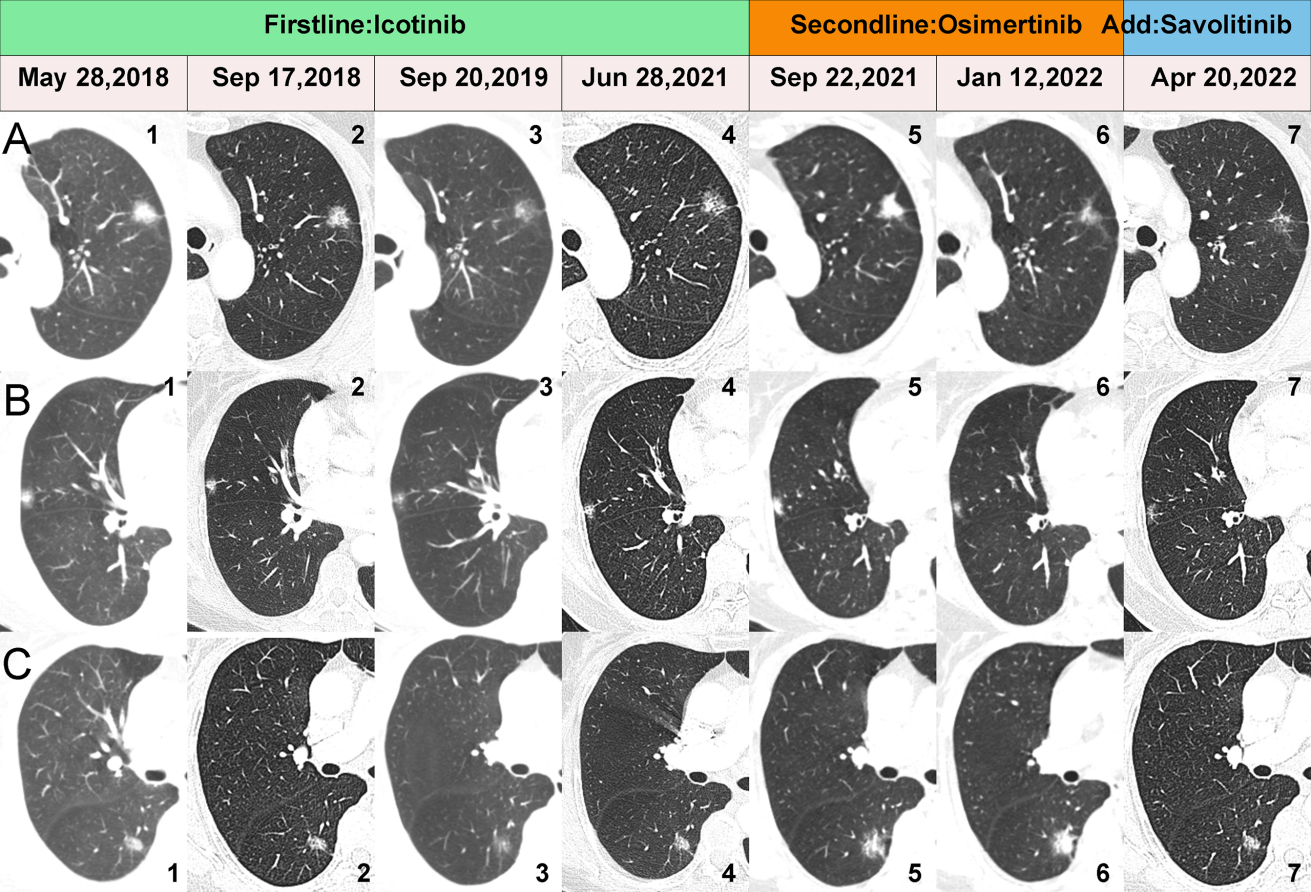
CT scans showing changes in the three lesions during the whole treatment. **(A1-7)** show the dynamic changes of the L1. **(B1-7)** show the dynamic changes of the L2. **(C1-7)** show the dynamic changes of the L3.

A 70-year-old female with no history of smoking sought treatment in our hospital on May 27, 2018 due to cough for 2 months. Her chest enhanced CT showed one subsolid node in each of the left upper lobe, right middle lobe, and right lower lobe (**Figures 1A1, B1, C1**). Due to her advanced age, her family members refused surgical treatment. Therefore, CT-guided biopsy was performed on all three nodules to clarify their pathological properties. In addition, targeted capture-based next-generation sequencing (NGS) of the punctured tissues was performed. The upper lobe of the left lung had adenocarcinoma (lepidic growth) with *EGFR* exon 19 deletion mutation (L1). The middle lobe of right lung had adenocarcinoma (acinar growth) with *EGFR* exon 19 deletion mutation (L2). The lower lobe of the right lung had adenocarcinoma (solid) accompanied by point mutation of *EGFR* exon 21, L858R (L3). The patient started oral icotinib 125 mg Q8H treatment on June 15, 2018, and the three cancer lesions all showed persistent good responses (**Figures 1A1-4, B1-4, C1-4**) until September 22, 2021, when re-examination by chest CT showed that all three lesions had grown and progressed (**Figures 1A5, B5, C5**).

**Figure 2:**
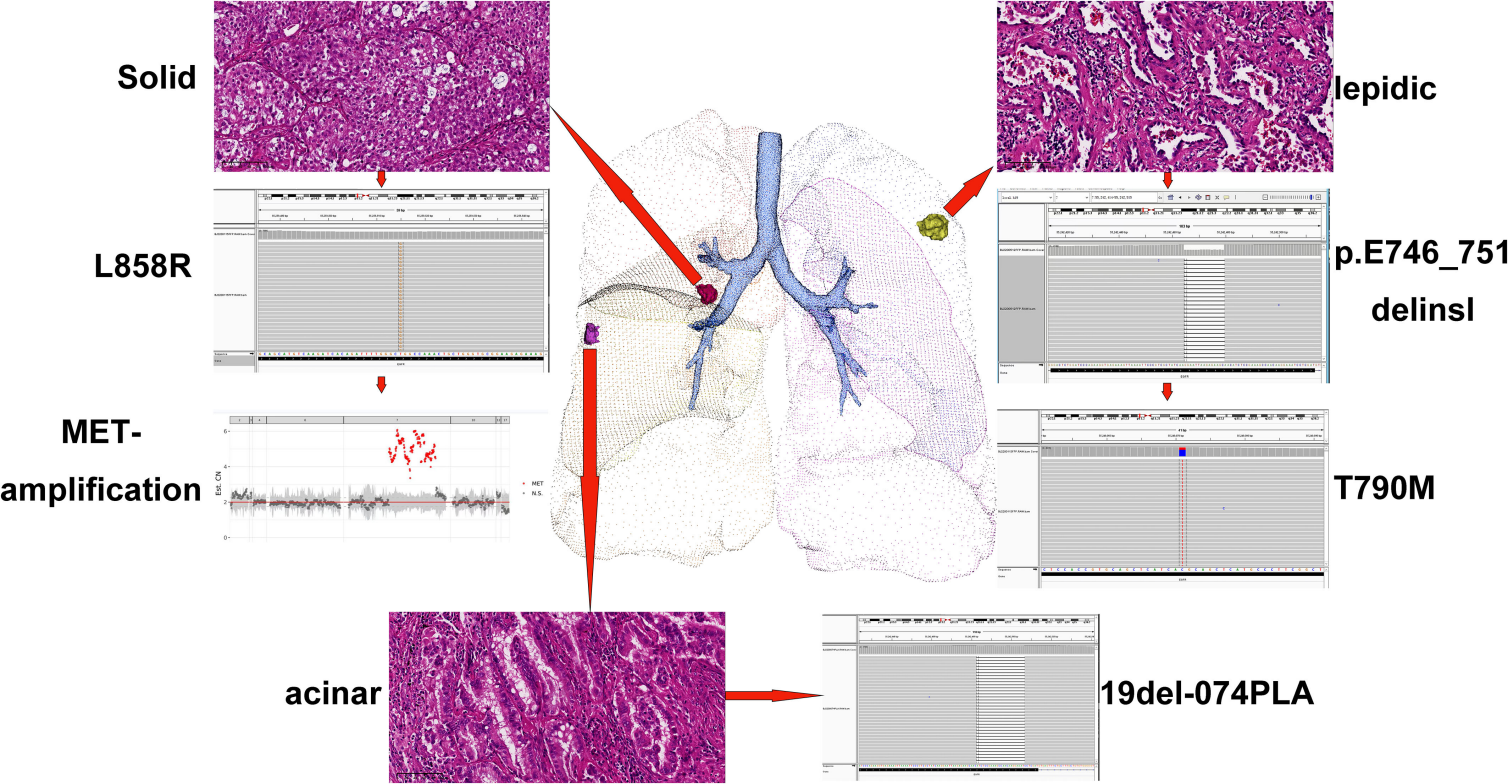
Location, pathology, and ongoing NGS-testing results of the three pulmonary lesions. NGS, next-generation sequencing.

To clarify the drug resistance mechanism of the EGFR-TKI in this patient, the left upper lobe lesion (L1) was punctured again, and peripheral venous blood of the patient was collected for NGS detection. Rebiopsy of the L1 tumor showed adenocarcinoma, And the L1 tumor tissue and peripheral venous blood tests all suggested *EGFR* exon 20 T790M mutation. Based on this result, the drug was changed to osimertinib orally at 80 mg qd on September 29, 2021. A CT reexamination on January 12, 2022 showed that the left upper lobe (L1) and the right middle lobe (L2) lesions had responded well to osimertinib, and the efficacy was evaluated as a partial response (**Figures 1A5-6, B5-6**). The efficacy in the right lower lobe lesion (L3) was evaluated as progressive disease (**Figures 1C5-6**). To investigate why L3 did not respond to osimertinib, the right lower lobe cancer lesion was punctured, and NGS detection was performed. Rebiosy of L3 tumor showed adenocarcinoma and the results of NGS suggested *MET* amplification. After multidisciplinary team discussion, the patient was given osimertinib combined with savolitinib. Chest CT reexamination on April 20, 2022 showed that the lesion in the right lower lobe was also well controlled, with an efficacy evaluated as partial response. Two other lesions also showed sustained responses (**Figures 1A6-7, B6-7, C6-7**). The location, pathology, and ongoing NGS-testing results of the three pulmonary lesions were showed in **Figure 2**.

## Discussion

The management of SMPLC should be based on the judgment of a multidisciplinary team, Surgical approach is the first choice recommended by the American College of Chest Physicians for those with SMPLC (10). Reports have shown that SMPLCs have a high incidence of driver mutations, such as EGFR mutations (6–8),implying an opportunity for targeted therapies in SMPLC management, especially in patient who can`t undergo surgery. However, some obstacles were observed. The tumor harboring a targetable mutation may not be representative of other lesion. The discrepancy rate of driver mutations in SMPLCs is relatively high, ranging from 72.0% to 92.1% (7–9). And like the other patients receiving target therapy, they face the problem of secondary drug resistance.

**Figure 3:**
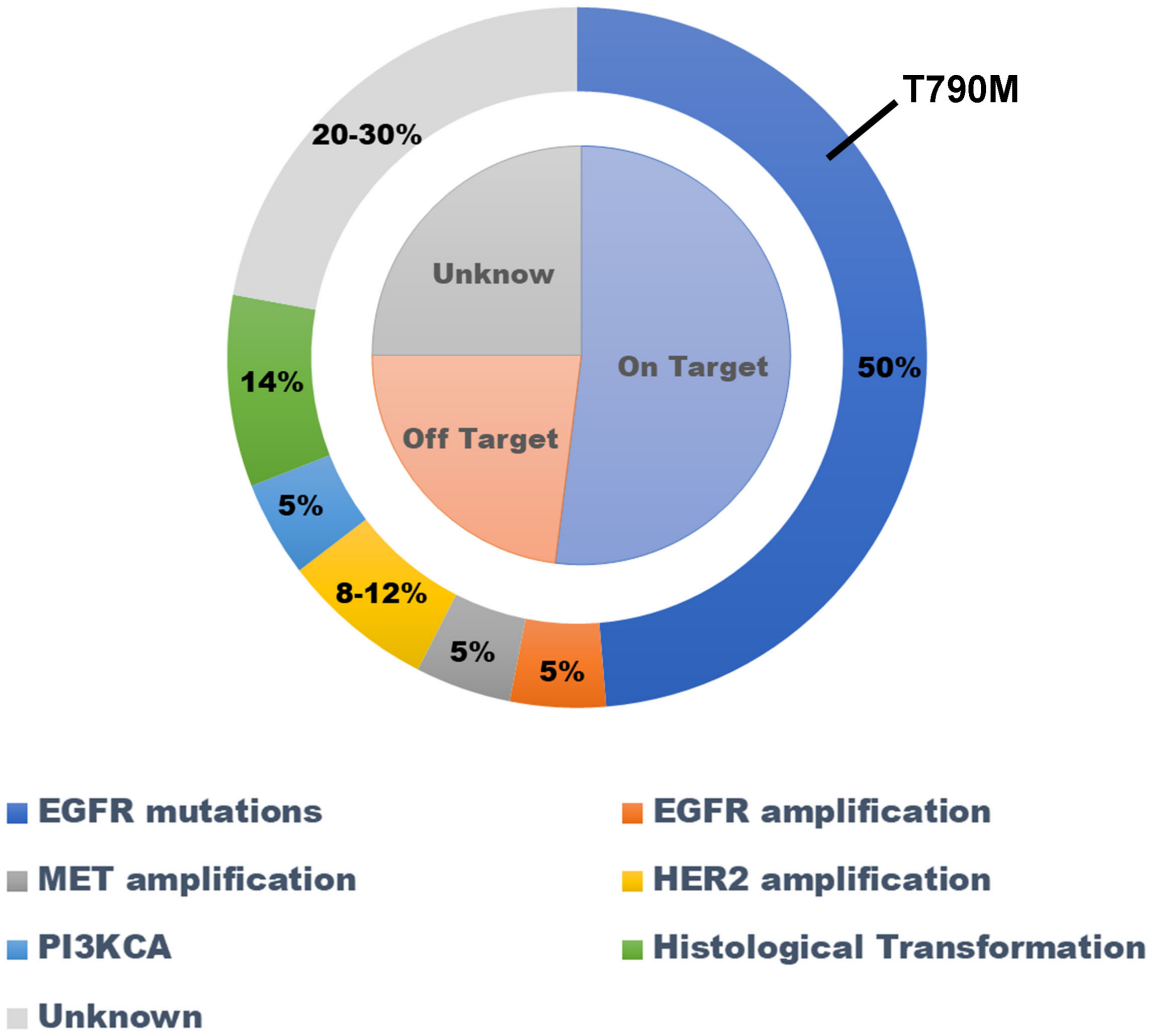
Mechanisms of drug resistance associated with using first generation EGRFR-TKI as first-line treatment for lung adenocarcinoma with *EGFR* mutation. EGFR-TKI, epidermal growth factor receptor–tyrosine kinase inhibitor.

The *EGFR* gene is the most common driver gene mutated in lung adenocarcinoma.EGFR-TKI is the preferred treatment for inoperable lung adenocarcinoma patients with *EGFR* mutations (11). All patients will develop secondary drug resistance after receiving EGFR-TKI treatment (12). The mechanisms of secondary drug resistance can be divided into EGFR-dependent mechanisms and non-EGFR-dependent mechanisms (**Figure 3**).

Here, we report a rare case of three primary lung adenocarcinoma lesions, each with an independent driver gene mutation. The left upper lobe lesion carried the *EGFR* 19 (p. E746_T751delinsl) mutation, the lesion in the middle lobe of the lung carried the *EGFR* 19Del-074PLA mutation, and the lesion in the lower lobe of the right lung carried the *EGFR* 21L858R mutation. After first-generation EGFR-TKI treatment, two lesions showed two different secondary drug resistance mechanisms. The lesions in the left upper lobe showed the T790M mutation, while the lesions in the right lower lobe showed *MET* amplification. At the same time, only T790M was detected by two NGS tests based on circulating tumor DNA (ctDNA), but not MET-amplification. The left upper lobe lesions showed a good response to osimertinib, while the right lower lobe lesions did not. After *MET* amplification was found in the right lower lobe lesion, savolitinib was added to oral osimertinib. The three lesions of the patient showed good responsiveness and tolerability.

CtDNA has emerged as an appealing approach that permits the minimally invasive genotyping of NSCLC (13). After the patient developed secondary drug resistance, we performed two NGS tests based on the patient`s ctDNA, accompanied by the second biopsy of L1 and L3,respectively, and only T790M was detected in both tests. This result may be consistent with Lam`s suggestion that TP53 and EGFR mutations are independent predictors of increased ctDNA shedding (14).Based on this situation, NGS testing based on ctDNA can`t replace NGS based on tissue for SMPLC patients with secondary drug resistance,even if a positive result occurs.

This is the first case report of SMPLC showing different drug resistance mechanisms after receiving first-generation EGFR-TKI treatment. This study provides novel ideas and experience for the diagnosis and treatment of SMPLC patients who cannot undergo surgical resection. Due to the differences in molecular biology of SMPLC, NGS technology should be utilized whenever possible to detect the molecular biology of patients’ lesions in order to guide their treatment.

At the same time, each lesion may also have different mechanisms of drug resistance to targeted therapy drugs. For such patients with multiple independent lesions, the lesions should be biopsied again if possible after the emergence of drug resistance to clarify the mechanism of drug resistance and to guide the targeted treatment. The application scenarios of ctDNA-based NGS detection need to be further studied.

## Data availability statement

The original contributions presented in the study are included in the article/supplementary material. Further inquiries can be directed to the corresponding author.

## Ethics statement

Ethical review and approval was not required for the study on human participants in accordance with the local legislation and institutional requirements. Written informed consent for participation was not required for this study in accordance with the national legislation and the institutional requirements. Written informed consent was obtained from the individual(s) for the publication of any potentially identifiable images or data included in this article.

## Author contributions

SX: Conceptualization, data curation, writing - original draft. QL: Conceptualization, funding acquisition, writing - review & editing.

## Funding

This work was supported by Medical science research project of Hebei province (Grant No. 20201076), the program of the government funding Clinical Excellence of Hebei province (2019Grant No. 139), and the program of the government funding Clinical Excellence of Hebei province (2022Grant No. 126).

## Conflict of interest

The authors declare that the research was conducted in the absence of any commercial or financial relationships that could be construed as a potential conflict of interest.

## Publisher’s note

All claims expressed in this article are solely those of the authors and do not necessarily represent those of their affiliated organizations, or those of the publisher, the editors and the reviewers. Any product that may be evaluated in this article, or claim that may be made by its manufacturer, is not guaranteed or endorsed by the publisher.

## References

[1] Siegel RL Miller KD Fuchs HE Jemal A. Cancer statistics, 2021. CA Cancer J Clin (2021) 71(1):7–33. doi: 10.3322/caac.21654 33433946

[2] Huo JW Luo TY He XQ Gong JW Lv FJ Li Q. Radiological classification, gene-mutation status, and surgical prognosis of synchronous multiple primary lung cancer. Eur Radiol (2022) 32(6):4264–74. doi: 10.1007/s00330-021-08464-x 34989846

[3] Chen TF Xie CY Rao BY Shan SC Zhang X Zeng B. Surgical treatment to multiple primary lung cancer patients: a systematic review and meta-analysis. BMC Surg (2019) 19(1):185. doi: 10.1186/s12893-019-0643-0 31795997 PMC6892192

[4] Wang M Herbst RS Boshoff C. Toward personalized treatment approaches for non-small-cell lung cancer. Nat Med (2021) 27(8):1345–56. doi: 10.1038/s41591-021-01450-2 34385702

[5] Yang CY Yang JC Yang PC. Precision management of advanced non-small cell lung cancer. Annu Rev Med (2020) 71:117–36. doi: 10.1146/annurev-med-051718-013524 31986082

[6] Yang Y Yin W He W Jiang C Zhou X Song X. Phenotype-genotype correlation in multiple primary lung cancer patients in China. Sci Rep (2016) 6:36177. doi: 10.1038/srep36177 27796337 PMC5087074

[7] Liu M He WX Song N Yang Y Zhang P Jiang GN. Discrepancy of epidermal growth factor receptor mutation in lung adenocarcinoma presenting as multiple ground-glass opacities. Eur J Cardiothorac Surg (2016) 50(5):909–13. doi: 10.1093/ejcts/ezw113 27032467

[8] Wu C Zhao C Yang Y He YY Hou LK Li XF. High discrepancy of driver mutations in patients with NSCLC and synchronous multiple lung ground-glass nodules. J Thorac Oncol (2015) 10(5):778–83. doi: 10.1097/JTO.0000000000000487 25629635

[9] Qu R Tu D Ping W Zhang N Fu X. Synchronous multiple lung cancers with lymph node metastasis and different EGFR mutations: Intrapulmonary metastasis or multiple primary lung cancers. Onco Targets Ther (2021) 14:1093–9. doi: 10.2147/OTT.S294953 PMC7896798 33623395

[10] Kozower BD Larner JM Detterbeck FC Jones DR. Special treatment issues in non-small cell lung cancer: Diagnosis and management of lung cancer, 3rd ed: American college of chest physicians evidence-based clinical practice guidelines. Chest (2013) 143(5 Suppl):e369S–99S. doi: 10.1378/chest.12-2362 23649447

[11] Shi YK Wang L Han BH Li W Yu P Liu YP. First-line icotinib versus cisplatin/pemetrexed plus pemetrexed maintenance therapy for patients with advanced EGFR mutation-positive lung adenocarcinoma (CONVINCE): a phase 3, open-label, randomized study. Ann Oncol (2017) 28(10):2443–50. doi: 10.1093/annonc/mdx359 28945850

[12] Jackman D Pao W Riely GJ Engelman JA Kris MG Jänne PA. Clinical definition of acquired resistance to epidermal growth factor receptor tyrosine kinase inhibitors in non-small-cell lung cancer. J Clin Oncol (2010) 28(2):357–60. doi: 10.1200/JCO.2009.24.7049 PMC3870288 19949011

[13] Aggarwal C Rolfo CD Oxnard GR Gray JE Sholl LM Gandara DR. Strategies for the successful implementation of plasma-based NSCLC genotyping in clinical practice. Nat Rev Clin Oncol (2021) 18(1):56–62. doi: 10.1038/s41571-020-0423-x 32918064

[14] Lam VK Zhang J Wu CC Tran HT Li L Diao L. Genotype-specific differences in circulating tumor DNA levels in advanced NSCLC. J Thorac Oncol (2021) 16(4):601–9. doi: 10.1016/j.jtho.2020.12.011 PMC8012216 33388476

